# Cannabidiol Modulates Mitochondrial Redox and Dynamics in MCF7 Cancer Cells: A Study Using Fluorescence Lifetime Imaging Microscopy of NAD(P)H

**DOI:** 10.3389/fmolb.2021.630107

**Published:** 2021-05-11

**Authors:** Rhys Richard Mould, Stanley W. Botchway, James R. C. Parkinson, Elizabeth Louise Thomas, Geoffrey W Guy, Jimmy D. Bell, Alistair V. W. Nunn

**Affiliations:** ^1^ Research Centre for Optimal Health, School of Life Sciences, University of Westminster, London, United Kingdom; ^2^ Central Laser Facility, Science and Technology Facilities Council, UKRI, Rutherford Appleton Laboratory, Harwell Campus, Oxford, United Kingdom; ^3^ GW Pharmaceuticals, Salisbury, United Kingdom

**Keywords:** cannabidiol, mitochondrial dynamics, imaging, redox, FLIM, NADH autofluorescence, multiphoton

## Abstract

The cannabinoid, cannabidiol (CBD), is part of the plant's natural defense system that when given to animals has many useful medicinal properties, including activity against cancer cells, modulation of the immune system, and efficacy in epilepsy. Although there is no consensus on its precise mode of action as it affects many cellular targets, CBD does appear to influence mitochondrial function. This would suggest that there is a cross-kingdom ability to modulate stress resistance systems that enhance homeostasis. As NAD(P)H autofluorescence can be used as both a metabolic sensor and mitochondrial imaging modality, we assessed the potential of this technique to study the *in vitro* effects of CBD using 2-photon excitation and fluorescence lifetime imaging microscopy (2P-FLIM) of NAD(P)H against more traditional markers of mitochondrial morphology and cellular stress in MCF7 breast cancer cells. 2P-FLIM analysis revealed that the addition of CBD induced a dose-dependent decrease in bound NAD(P)H, with 20 µM treatments significantly decreased the contribution of bound NAD(P)H by 14.6% relative to the control (*p* < 0.001). CBD also increased mitochondrial concentrations of reactive oxygen species (ROS) (160 ± 53 vs. 97.6 ± 4.8%, 20 µM CBD vs. control, respectively, *p* < 0.001) and Ca^2+^ (187 ± 78 vs. 105 ± 10%, 20 µM CBD vs. the control, respectively, *p* < 0.001); this was associated with a significantly decreased mitochondrial branch length and increased fission. These are all suggestive of mitochondrial stress. Our results support the use of NAD(P)H autofluorescence as an investigative tool and provide further evidence that CBD can modulate mitochondrial function and morphology in a dose-dependent manner, with clear evidence of it inducing oxidative stress at higher concentrations. This continues to support emerging data in the literature and may provide further insight into its overall mode of action, not only in cancer, but potentially its function in the plant and why it can act as a medicine.

## Introduction

Uncontrolled cell growth, or cancer, is frequently associated with increased aerobic glycolysis (the Warburg effect) and alterations in mitochondrial function (Trigos et al., 2018). A plant’s ability to develop tumors could explain why so many secondary plant phenolic compounds appear to have anticancer activity in both plant and animal models (Rasouli et al., 2016); over 3,000 species of plants have anticancer activity in animals, with many modulating mitochondrial function and apoptosis (Gali-Muhtasib et al., 2015). Due to the similarities between plant and animal metabolism, many plant compounds can be considered mitochondrially targeted drugs for treating cancer (Gorlach et al., 2015). In plants, mitochondria are central in managing oxidative stress *via* thermodynamic buffering, involving enhancement of antioxidant capacity and uncoupling, as well as detoxification and induction of programmed cell death (Popov, 2020; Gandin et al., 2021). This is supported by evidence that some bioactive plant compounds can protect plant mitochondria (Laus and Soccio, 2020). Critically, the stress response system has to not only be adaptive and protect cells but also induce cell death when necessary, suggesting a biphasic or hormetic dose–response curve. One such compound, cannabidiol (CBD), has demonstrated a plethora of pharmacological effects ranging from anti-inflammatory to anxiolytic, antiepileptic, anticancer, and even antibacterial (Mechoulam et al., 2002; Gray et al., 2020; Kis et al., 2019). However, there is still no clear consensus on its mode of action. Rather than focusing on individual receptors and channels, here we investigate how it might modulate a key nexus in cell function, the mitochondrion.

CBD, along with Δ9-tetrahydrocannabinol (THC), is a major phytocannabinoid and both are well described components of medicines. Unlike THC, CBD is not psychoactive and is now being used to treat epilepsy (O’Connell et al., 2017; Baker et al., 2000). A growing number of studies have demonstrated the anticancer properties of CBD, in both *in vitro* and *in vivo* models (Ramer and Hinz, 2017; Kis et al., 2019). Proposed mechanisms include induction of autophagy and mitochondrial-mediated apoptosis and inhibition of exosome and microvesicle release (Shrivastava et al., 2011; Armstrong et al., 2015; McAllister et al., 2015; Kosgodage et al., 2018). Although the exact cellular mechanisms by which CBD exerts its effects remain unclear (Ibeas Bih et al., 2015), one potential mechanism may relate to its ability to interact with the voltage-dependent anion channel 1 (VDAC1) (Rimmerman et al., 2013), which is central to metabolic reprogramming, apoptosis, and a cancer drug target (Shoshan-Barmatz et al., 2018). VDAC1 is also important in controlling mitochondrial morphology as part of the mitochondrial-associated membrane (MAM) complex and a key controller of calcium flux (Giorgi et al., 2015). Critically, cancer cells modulate mitochondrial dynamics to resist apoptosis (Senft and Ronai, 2016). We have preliminary data that indicate that CBD modulates mitochondrial dynamics (Nunn et al., 2013), supported by recent studies which suggest CBD modulates the membrane order and induces cholesterol biosynthesis (Guard et al., 2020).

Another important target for CBD, the transient receptor potential vanilloid 1 (TRPV1) (Bisogno et al., 2001; Iannotti et al., 2014) is critical in calcium signaling and can lead to its accumulation by mitochondria (Bujak et al., 2019). CBD stimulates neuronal survival in models of Parkinson’s disease via the protein kinase B/mammalian target of rapamycin (AKT/mTOR) pathway through a mechanism involving TRPV1 (Gugliandolo et al., 2020) and can also induce cancer cell death by inhibiting AKT/mTOR (Pisanti et al., 2017). Similarly, CBD reduces oxidative stress, enhances mitochondrial function, and stimulates G6PDH activity and the pentose phosphate pathway (PPP) in cerebral ischemia (Sun et al., 2017). However, enhancing PPP pathway components in mitochondria, such as G6PDH could, by reducing oxidative stress, suppress apoptosis and thus enhance oncogenesis (Redhu and Bhat, 2020). This would begin to explain why a single compound can both be protective (e.g., in neurons) and capable of killing cancer cells.

It is possible that CBD directly modulates mitochondrial function as part of its mode of action. Balance between reduced nicotinamide adenine dinucleotide (NADH) and nicotinamide adenine dinucleotide phosphate (NADPH) plays a key role in balancing oxidative stress. This, in concert with markers such as reactive oxygen species (ROS), calcium, and mitochondrial dynamics could be informative in confirming this. In recent years, 2P-FLIM of the reduced forms of NADH and NADPH has emerged as a viable means of assessing the redox state of the cell (Yaseen et al., 2017) and is used to identify numerous pharmacological, physiological, and pathophysiological disruptions in NAD^+^/NADH and NADP^+^/NADPH metabolism (Yu and Heikal, 2009; Stringari et al., 2011; Skala et al., 2007a). The spectral properties of NADH and NADPH are indistinguishable and are therefore referred to as NAD(P)H. Moreover, NAD(P)H fluorescence is an effective way of imaging mitochondria, as NADH is predominantly found in the mitochondrion (Yu and Heikal, 2009).

NADH is a central cofactor in both anaerobic glycolysis and aerobic oxidative pathways and plays a critical role in controlling cellular energy production (Blacker and Duchen, 2016). The intrinsic fluorescence of NAD(P)H enables two photon fluorescence lifetime imaging (2P-FLIM) to differentiate between components of free or bound NAD(P)H species, thereby reflecting changes in enzymatic function and representing a potential label-free, direct marker of cellular metabolism. The excited state lifetime of a molecule allows for the extraction of additional information, as it is determined by a wide range of environmental parameters, including oxygen concentration, pH, ions, molecular binding, and the proximity to other molecules, making it currently the technique of choice for functional imaging in live cells and tissue. The use of two-photon excitation also provides further advantages; for example, using excitation wavelengths in the near infra-red (NIR) that are tissue-friendly compared to ultraviolet radiation, decreasing overall cell and tissue autofluorescence and light scattering, and improved tissue depth penetration which allows diffraction limited imaging selectivity, noninvasively confined to the focal volume (Suhling et al., 2015).

Here we report the use of FLIM to further assess the effects of CBD on mitochondrial metabolism and morphology in MCF7 breast adenocarcinoma cells. To achieve this, we used NAD(P)H autofluorescence by applying 2P-FLIM to provide a label-free marker of the metabolic state and mitochondrial morphology and more conventional fluorescent markers to study ROS and mitochondrial calcium levels.

## Materials and Methods

### Cell Culture

MCF7 cells (from ATCC, provided by Central Laser Facility, UKRI) were cultured in minimal essential media (MEM), supplemented with 10% FBS and 1% penicillin/streptomycin. For the experiments, cells were seeded onto individual dishes or multiwell plates and treated with 0.005% v/v DMSO (Control) or 1, 5, 10, and 20 µM CBD (Sigma, United Kingdom) for 24 h prior to assessment at 37°C with 5% CO_2_ humidified air.

### 2-Photon Fluorescence Lifetime Imaging Microscopy

2P-FLIM was used to image and quantify NAD(P)H lifetime decay as a measure of cellular and mitochondrial metabolism. MCF7 cells were seeded at densities of 1.5 × 10^5^ onto individual glass bottom dishes (MatTek) and treated as described. Before imaging, cells were acclimatized for 15 min to room temperature. The 2P-FLIM setup has been described previously (Botchway et al., 2015). Images were obtained as follows: 750 nm wavelength light for 2-photon excitation of NADH was generated by a mode-locked titanium sapphire laser (Mira F900, Coherent Laser Ltd.), producing 180 fs pulses at 76 MHz. This laser was pumped by a solid-state continuous wave 532 nm laser (Verdi 18, Coherent Lasers Ltd.). Images were collected through a water immersion 60X 1.2 NA objective on a modified Nikon EC2 confocal scanning system attached to a TE2000-U microscope as described previously (Botchway et al., 2015). Emission was collected by the same objective, reflected off a 530 BK-25 filter (Comar Optics, United Kingdom), passed through a BG39 filter and detected with an external hybrid GaAsP (HPM-100–40, Becker and Hickl, Germany), linked to a time correlated single photon counting (TCSPC) module (SPC830, Becker and Hickl, Germany). In this configuration, together with an average laser power of 0.5 mW at the sample, we mostly observe the fluorescence signal from NAD(P)H with negligible contribution from the flavins. This was confirmed with solution phase studies of NAD(P)H and FAD (both purchased from Sigma, United Kingdom, and used without further purification). We have performed a dual channel FLIM setup containing BG39 with or without extra band pass filters (a 440–490 nm band pass filter for NADH and a 520–570 nm band pass filter for FAD and flavins), and we did not detect flavin emission in the NADH channel. Solution concentrations as high as 0.5 mM are observed in the flavin-detecting channel only. The intracellular FAD concentration has been estimated to be around 8 μM (Kimata et al., 2018). For each of the five experimental groups (Control, 1, 5, 10, and 20 μm CBD), five spatially distinct 2P-FLIM images were acquired. Within each image, five nonoverlapping cells with the highest signal-to-noise ratio were chosen, equating to 25 cells per treatment group. As NAD(P)H signal intensity aligns with mitochondrial morphology (See Supplementary Figure S1), pixels corresponding to the mitochondria or the cytosol could be manually selected (See Supplementary Figure S2) (the spatial resolution of the setup was determined to be ∼350 nm) (Alam et al., 2017). Photon counts of at least 1,000 were used for the multiexponential analysis, which also excluded nuclear areas from analysis. The decay curve of each pixel from the 2P-FLIM image was modeled with a biexponential decay curve, according to Eq. 1, where I(t) represents fluorescence intensity at time t after the laser pulse. Goodness of fit was quantified by Chi-squared analysis (See Supplementary Figure S3). From each pixel two observed lifetimes were recorded: τ_Free_ for free NAD(P)H and τ_Bound_ for enzyme-bound NAD(P)H and the contributions of each lifetime to the overall decay curve, α_Free_ and α_Bound_, respectively (Sharick et al., 2018; Blignaut et al., 2019).

$$I\left(t\right)=\;\alpha_{Free}exp^{-t/\tau_{1}}+\alpha_{Bound}exp^{-t/\tau_{2}}\;$$ [1]

### Mitochondrial Reactive Oxygen Species and Ca^2+^

Mitochondrial ROS and Ca^2+^ levels were quantified using the MitoSOX Red (ThermoFisher, United Kingdom) and Rhod2 (ThermoFisher, United Kingdom) stains, respectively, in octuplicate, as per manufacturer’s instructions. Briefly, MCF7 cells were seeded at densities of 3.0 × 10^4^ cells per well. Following treatment, cells were stained with either 5 µM MitoSOX Red or 1 µM Rhod2 for 30 min. Fluorescence was measured on a FLUOstar Optima microplate reader (BMG, Germany). Excitation/Emission: 510/580 nm for MitoSOX Red and 552/580 nm for Rhod2. Fluorescence intensities were normalized to total cellular protein, measured using the Bradford assay.

### Mitochondrial Morphology Analysis

Mitochondrial morphology was analyzed as previously described (Valente et al., 2017). Briefly, MCF7 cells were seeded at densities of 1.0 × 10^5^ cells per well and treated with DMSO (Control) or CBD for 24 h as described above. Following treatment, cells were stained with MitoTracker Deep Red and NucBlue (ThermoFisher, United Kingdom) and imaged using the ThermoFisher EVOS FL2 Auto using a 40X 0.65 NA plan fluorite objective. Five representative images were selected per treatment group and within each image, the mitochondria of three spatially distinct cells were selected for analysis. To quantify CBD-induced changes in morphology, the cells were analyzed with the MiNA plugin for ImageJ. This script, following a preprocessing stage of unsharp masking, local contrast enhancement (CLAHE), and median filtering, binarized and then skeletonized the mitochondrial image to produce an output of the mitochondrial footprint (the total area of the binary image), mean length of mitochondrial networks, mean branch length per network (the mean of the sum length of individual branches on separate networks) and mean number of networks. Three cells were analyzed per image for the five images. This experiment was carried out in triplicate.

### Statistics

Data are presented as box plots, the box shows median with 25th and 75th percentiles, and the whiskers indicate minimum and maximum values. Statistical significance was calculated by one-way ANOVA with Dunnett’s multiple comparison using GraphPad Prism version 8.0 (GraphPad Software, La Jolla, California, United States). An adjusted *p* < 0.05 was considered statistically significant.

## Results

**FIGURE 1:**
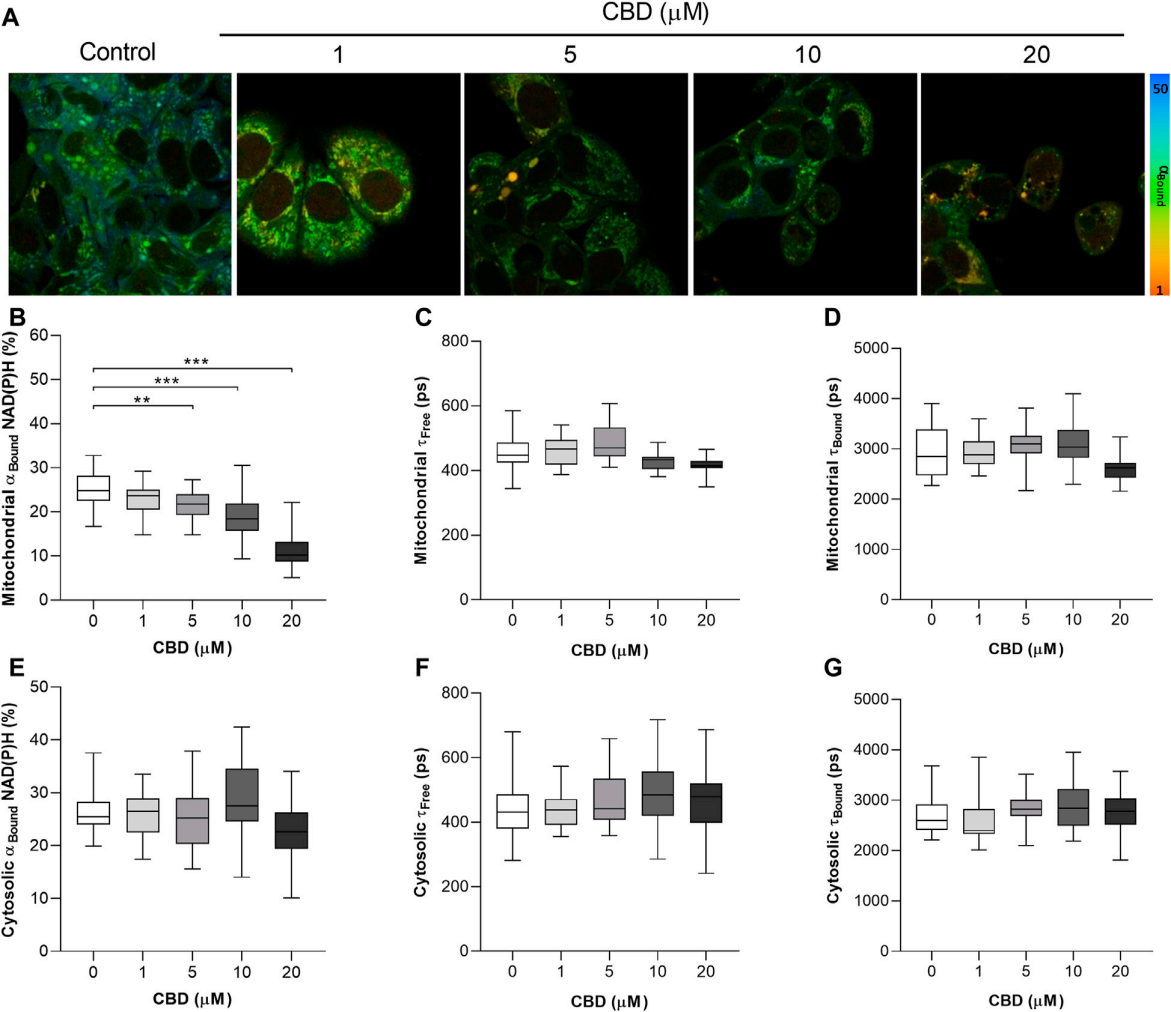
*In vitro* effects of cannabidiol on NAD(P)H fluorescence decay as measured by 2P-FLIM. **(A)** 2P-FLIM–derived images of bound NAD(P)H (α_Bound_). **(B–G)** Box plots showing the effect of CBD on **(B)** mitochondrial α_Bound_ NAD(P)H (%), **(C)** mitochondrial τ_Free_ (ps), **(D)** mitochondrial τ_Bound_ (ps), **(E)** cytosolic α_Bound_ NAD(P)H (%), **(F)** cytosolic τ_Free_ (ps), and **(G)** cytosolic τ_Bound_. Box shows median with 25th and 75th percentiles, and whiskers indicate minimum and maximum values. *n* = 25/group; * = *p* < 0.05, ** = *p* < 0.01, *** = *p* < 0.001; data were analyzed by one-way ANOVA with Dunnett’s test for multiple comparisons using GraphPad Prism (United States).

The significant dose-dependent effects of CBD on the mitochondrial NAD(P)H lifetime are shown in Figure 1. Figure1A are representative 2P-FLIM–derived images of bound NAD(P)H in MCF7 cells 24 h post-treatment with CBD. Each pixel represents the contribution of bound NAD(P)H to the overall fluorescence decay, α_Bound,_ and is color-coded between 50% (blue) and 1% (red). The images indicate a shift from high (blue-green) to lower α_Bound_ (yellow-orange) as the concentration of CBD increases. Quantitative changes in α_Bound_ are shown in Figure 1B. CBD was found to induce a dose-dependent decrease in α_Bound_ (Control: 25.3 ± 3.9% vs CBD 20 μM: 11.3 ± 4.1%, *p* < 0.001). CBD had no significant effect on the fluorescence lifetimes of mitochondrial free NAD(P)H (τ_Free_) and bound NAD(P)H (τ_Bound_), shown in Figures 1C and D, respectively. The mean values for each are consistent with the literature that describe free NAD(P)H as having a shorter lifetime of approximately 0.4 ns and bound NAD(P)H with a longer lifetime of approximately 2.4 ns. No significant effect of CBD was observed on cytosolic levels of α_Bound_ NAD(P)H (Figure 1E). CBD had no significant effect on the fluorescence lifetimes of cytosolic τ_Free_ and τ_Bound_ (shown in Figures 1F and G, respectively), and this again aligns with the literature describing the lifetimes of each species.

**FIGURE 2:**
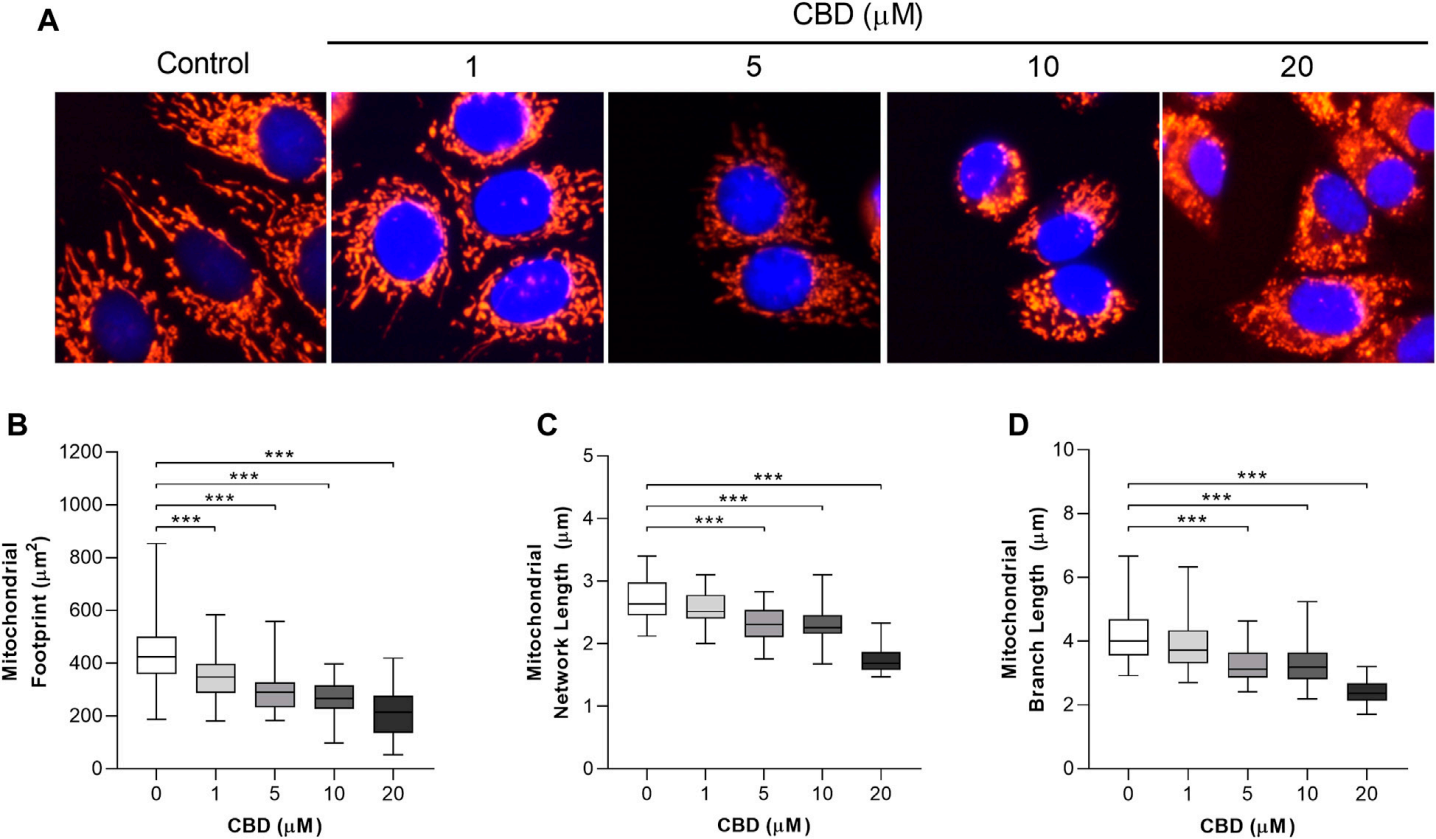
*In vitro* effects of cannabidiol on mitochondrial morphology. **(A)** Representative images showing changes in mitochondrial morphology 24 h posttreatment with CBD (1, 5, 10, and 20 µM) or the control (DMSO). Cells were stained with MitoTracker Deep Red and NucBlue (ThermoFisher, United Kingdom) and imaged using the ThermoFisher EVOS FL2. **(B–D)** Box plots showing the effect of CBD on **(B)** mitochondrial footprint, **(C)** mitochondrial network length, and **(D)** mean mitochondrial branch length. Box shows median with 25th and 75th percentiles, and whiskers indicate minimum and maximum values. n = 45/group; *** = *p* < 0.001; data were analyzed by one-way ANOVA with Dunnett’s test for multiple comparisons using GraphPad Prism (United States).

The *in vitro* effects of CBD on mitochondrial morphology in MCF7 cells are shown in Figure 2. Representative images are shown in Figure 2A. To quantify these changes in mitochondrial morphology we applied the MiNA plugin to assess the effects of CBD on mitochondrial area (Figure 2B), network length (Figure 2C), and branch length (Figure 2D). CBD was found to induce a dose-dependent decrease in the mitochondrial footprint (control: 436 ± 133 µm^2^ vs. CBD 20 μM: 214 ± 86 µm^2^, *p* < 0.001, Figure 2B), mean network length (control: 2.7 ± 0.3 µm vs. CBD 20 μM: 1.8 ± 0.2 µm, *p* < 0.001, Figure 2C), and mean branch length (control: 4.2 ± 0.7 µm vs. CBD 20 μM: 2.4 ± 0.4 µm, *p* < 0.001, Figure 2D).

**FIGURE 3:**
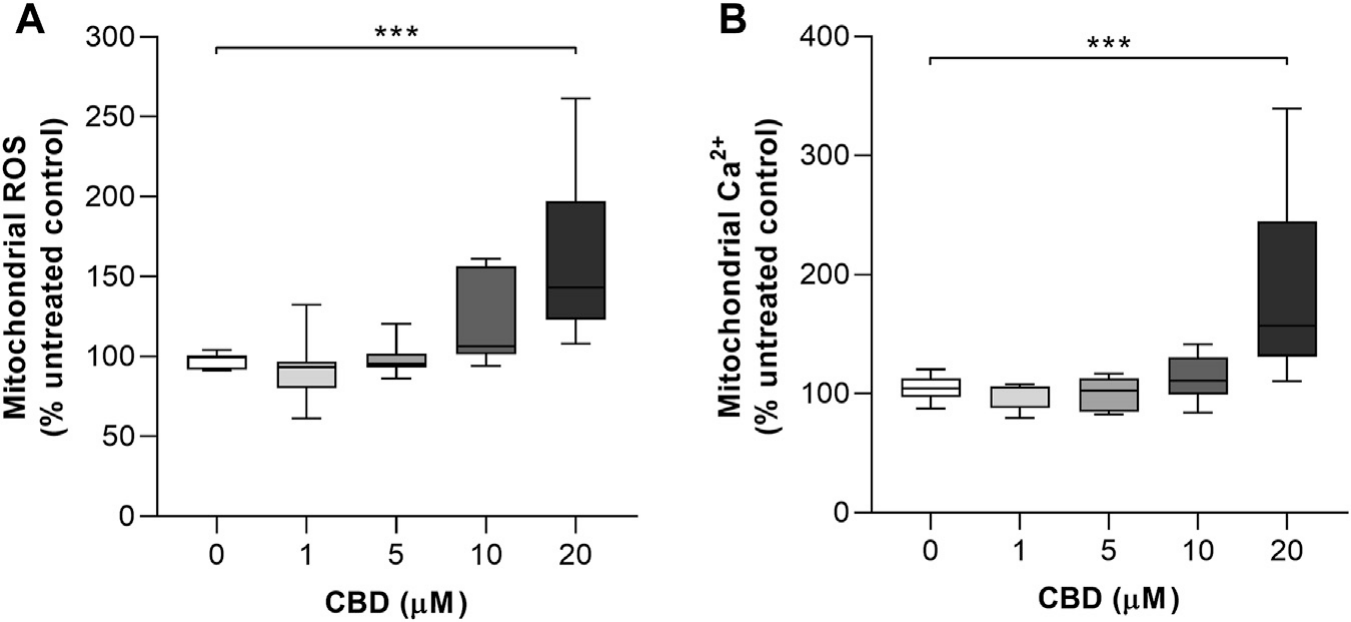
*In vitro* effects of CBD on mitochondrial ROS and mitochondrial Ca^2+^ production in MCF7 cells. **(A)** Effects of cannabidiol (CBD) on normalized MitoSOX intensity as a marker of mitochondrial reactive oxygen species (ROS) production. **(B)** Effects of CBD on normalized Rhod2 intensity as a marker of mitochondrial Ca^2+^ production. Box shows median with 25th and 75th percentiles, and whiskers indicate minimum and maximum values. n = 8/group; ** = *p* < 0.01, *** = *p*< 0.001; data were analyzed by one-way ANOVA with Dunnett’s test for multiple comparisons using GraphPad Prism (United States).

The effects of CBD treatment on mitochondrial ROS and mitochondrial Ca^2+^ levels are shown in Figures 3A and B, respectively. Compared to the controls, the highest dose of CBD significantly increased the mitochondrial levels of both ROS (Control: 97.6 ± 4.8% vs. CBD 20 μM: 160 ± 53, *p* < 0.001, Figure 3B) and Ca^2+^ (Control: 104.6 ± 10.3% vs. CBD 20 μM: 186.9 ± 78.2%, *p* < 0.01, Figure 3C).

## Discussion

In this study we used FLIM, fluorescence microscopy, and conventional ROS and calcium detecting stains to investigate the effects of CBD on NAD(P)H lifetime, calcium and ROS levels, and mitochondrial morphology in a breast cancer cell line. This study supports the use of FLIM to investigate how CBD may be working. The observed increase in mitochondrial ROS and calcium and enhanced mitochondrial fission and changes in bound NAD(P)H weighting are suggestive of CBD affecting mitochondrial function.

Studying mitochondrial function may be useful in explaining the beneficial actions of many natural products. This is reflected by the link between mitochondrial morphology and function in both immune cells (Rambold and Pearce, 2018) and cancer (Chen and Chan, 2017). Critically, many compounds that can suppress immunity are also antiproliferative; for instance, rapamycin, which modulates mTOR, is well known to be activated in cancer (Rad et al., 2018) and a modulator of mitochondrial function (Wei et al., 2015). In addition to metabolic reprogramming, mitochondria play a central role in regulating cell death, and resistance to proapoptotic signaling is a hallmark of cancer (Sayers, 2011).

### Understanding Warburg to Understand Cannabidiol

A paradox in the understanding of the function of plant products, including CBD, is how they can enhance survival of normal cells, yet kill cancer cells. The Warburg shift, or aerobic glycolysis (Kroemer, 2006; Wallace, 2012), may be key, as it is overall antiapoptotic and probably key in the earlier stages of carcinogenesis, as more advanced cancers often upregulate mitochondrial function. This has led to the so called “Inverse Warburg Hypothesis”, which, although not fully understood, suggests that high levels of mitochondrial metabolism underlie the observed inverse relationship between degenerative diseases and cancer. Mitochondria are pivotal in the detection of and adaptation to stress, sending out redox signals in response to stress, which may be an important mechanism explaining how plant secondary metabolites work as medicines via a process called mitohormesis (Tapia, 2006). Critically, evidence is accumulating that major plant stress signaling compounds, such as jasmonates, can modulate mitochondria in both plant and animal cells (Bömer et al., 2020). Moreover, plant compounds are known to interfere with glycolytic pathways and major controllers of metabolism, such as VDAC. Thus, they appear to inhibit carcinogenesis, but if a tumor does arise, they can alter its mitochondrial phenotype to restore cell death (Stevens et al., 2018).

### Known Mitochondrial Effects for Cannabidiol–Controlling Calcium Flux

In cancer cells, reduced endoplasmic reticulum–mitochondria Ca^2+^ exchange contributes to apoptotic resistance; increased intracellular Ca^2+^ contributes to enhanced cell migration, facilitating metastasis (Marchi and Pinton, 2016). This is matched by changes in mitochondrial morphology, making cells more resistant to apoptosis (Senft and Ronai, 2016). Critically, mitochondrial function is tightly controlled by calcium, ranging from stimulation to enhance respiration, to overload to induce cell death (Rossi et al., 2019)—and in cancer, calcium flow is directed away from the mitochondrion (Danese et al., 2017).

CBD modulates TRPV1 and calcium influx into the cell and potentially into the mitochondrion (Ryan et al., 2009; Mato et al., 2010; Olivas-Aguirre et al., 2019). More directly, CBD has been shown to bind to and close VDAC1, a major channel responsible for transporting Ca^2+^, potentially responsible for the immunosuppressive and anticancer effects of CBD (Rimmerman et al., 2013). In leukemic cells, CBD induces dose-related mitochondrial dysfunction, leading to apoptosis, which is associated with mitochondrial calcium overload, loss of mitochondrial membrane potential, and enhanced ROS production (Olivas-Aguirre et al., 2019). This all supports earlier data that CBD application can result in mitochondrial calcium overload and enhanced ROS production (Ryan et al., 2009; Mato et al., 2010).

### Interpreting the FLIM Data

In response to CBD, we identified a dose-dependent decrease in mitochondrial bound NAD(P)H (α_Bound_). This appears to indicate reduced activity of the ETC, as the oxidation of NADH by complex I is the first point where electrons enter the ETC (Chakraborty et al., 2016). Indeed, comparable reductions in NAD(P)H α_Bound_ have been observed in response to known mitochondrial toxins and inhibitors such as potassium cyanide and rotenone (Bird et al., 2005; Schneckenburger et al., 2004), while in contrast, agents which increase metabolic activity increase mitochondrial α_Bound_ NAD(P)H (Alam et al., 2017). So, in relation to our data, the increase in unbound NAD(P)H may represent an inhibition of the ETC and/or enhanced TCA leading to increased NADH, possibly coupled to inhibition of glycolysis and enhanced PPP activity, a generalized inhibition of metabolism leading more unbound NAD(P)H, and possibly, inhibition of NADP^+^ binding to G6PDH. The latter effect is a known ability of the polyphenol epigallocatechin gallate (EGCG) (Shin et al., 2008) which also modulates mitochondrial function (Oliveira et al., 2016). Inhibition of G6PD has also been observed for other phenolic compounds (Adem et al., 2014), while many have also been found to dissociate hexokinase from VDAC1, which inhibits glycolysis and can enhance apoptosis (Tewari et al., 2015; Tewari et al., 2017; Goldin et al., 2008). There is thus, perhaps, a generalization that many of these bioactive plant compounds have the capacity to induce metabolic reprogramming, which may also apply to CBD. Whilst the contribution of each species to the decay curve were affected by CBD treatment, there were no significant changes in the lifetimes of each species itself (τ_free_ and τ_Bound_). Changes in τ_Bound_ are often associated with a redistribution of NAD(P)H binding sites as a result of a shift in metabolic pathways (Skala et al., 2007b). The absence of changes in this study may indicate no such pivot in specific metabolic pathways, although further studies comparing CBD-mediated τ_free_ and τ_Bound_ to agents known to perturb mitochondrial metabolism would be required to validate this.

In terms of intracellular localization, 2P-FLIM permits highly localized, label-free, sub-micron determination of the redox state, enabling the differentiation of the mitochondrial redox response from that of the cytosol. The lack of effect on free or bound cytosolic NAD(P)H in our results would seem to indicate that the actions of CBD are mitochondrial specific, which has been proposed based on the highly lipophilic properties of CBD (Huestis, 2007; Ryan et al., 2009). However, the subtle, interconnected relationships between the many cellular pathways maintaining stores of NADH are not fully deconvoluted in the aggregate FLIM signal (Blacker and Duchen, 2016), making full interpretation difficult. For example, while cancer cells shift their preferred energy-generating pathway to aerobic glycolysis (Trigos et al., 2018), the relative rates of glycolysis and OXPHOS do not appear to affect the intracellular NADH fluorescence lifetime (Blacker and Duchen, 2016; Guo et al., 2015).

### Insight Into Mode of Action

It should be noted that the 24 h post‐exposure protocol employed here was based on our earlier investigations into the actions of CBD (Kosgodage et al., 2018) and therefore limits our ability to define an accurate timescale for the specific cascade of intracellular events following CBD administration. However, previous studies have indicated that CBD-triggered increases in Ca^2+^ precede any mitochondrial changes in ROS, membrane potential, or morphology (Olivas-Aguirre et al., 2019). This might suggest a sequential modulation of various parts of the cells, starting on the outside and working inwards as this highly lipophilic compound is absorbed by the cell.

Overall, our FLIM data suggest that CBD induces mitochondrial oxidative stress in these cancer cells, which correlate with the dose-related increase in mitochondrial ROS and calcium and evidence of increased mitochondrial fission. Although cancer cells often display abnormal mitochondrial dynamics, mitochondrial fission is often a response to oxidative stress, especially if ROS originates from damaged mitochondria and is associated with a loss of the mitochondrial membrane potential and influx of calcium, leading to mitophagy and renewal or cell death (Sharma et al., 2019). Mitochondrial fission can also form part of a positive feedback cytosolic calcium signaling pathway that can promote autophagy, which also seems to be reliant on increased ROS production (Huang et al., 2019). Mitochondria have a pivotal role in maintaining cellular homeostasis by sequestering and releasing intracellular Ca^2+^. Within the mitochondria, Ca^2+^ biphasically affects energy metabolism via activation/inhibition of mitochondrial enzymes and direct charge alteration of the mitochondrial membrane potential, which in turn regulates the supply of electrons into the respiratory chain and production of ROS (Danese et al., 2017). Ultimately however, prolonged accumulation of mitochondrial calcium, known as calcium overload, is associated with the mitochondrial permeability transition (MPT), a key step in the initiation of apoptosis, and has been identified in T-cells following treatment with CBD (Olivas-Aguirre et al., 2019). Indeed, these data support our previous observations of CBD-induced mitochondrial fission in a different cell line (Nunn et al., 2013) and our unpublished observations in several other cell lines. Overall, this supports the concept that CBD can induce ROS in a range of tissue types, including monocytes (Wu et al., 2018), glioma stem cells (Singer et al., 2015), and breast cancer cells (Kis et al., 2019).

**FIGURE 4:**
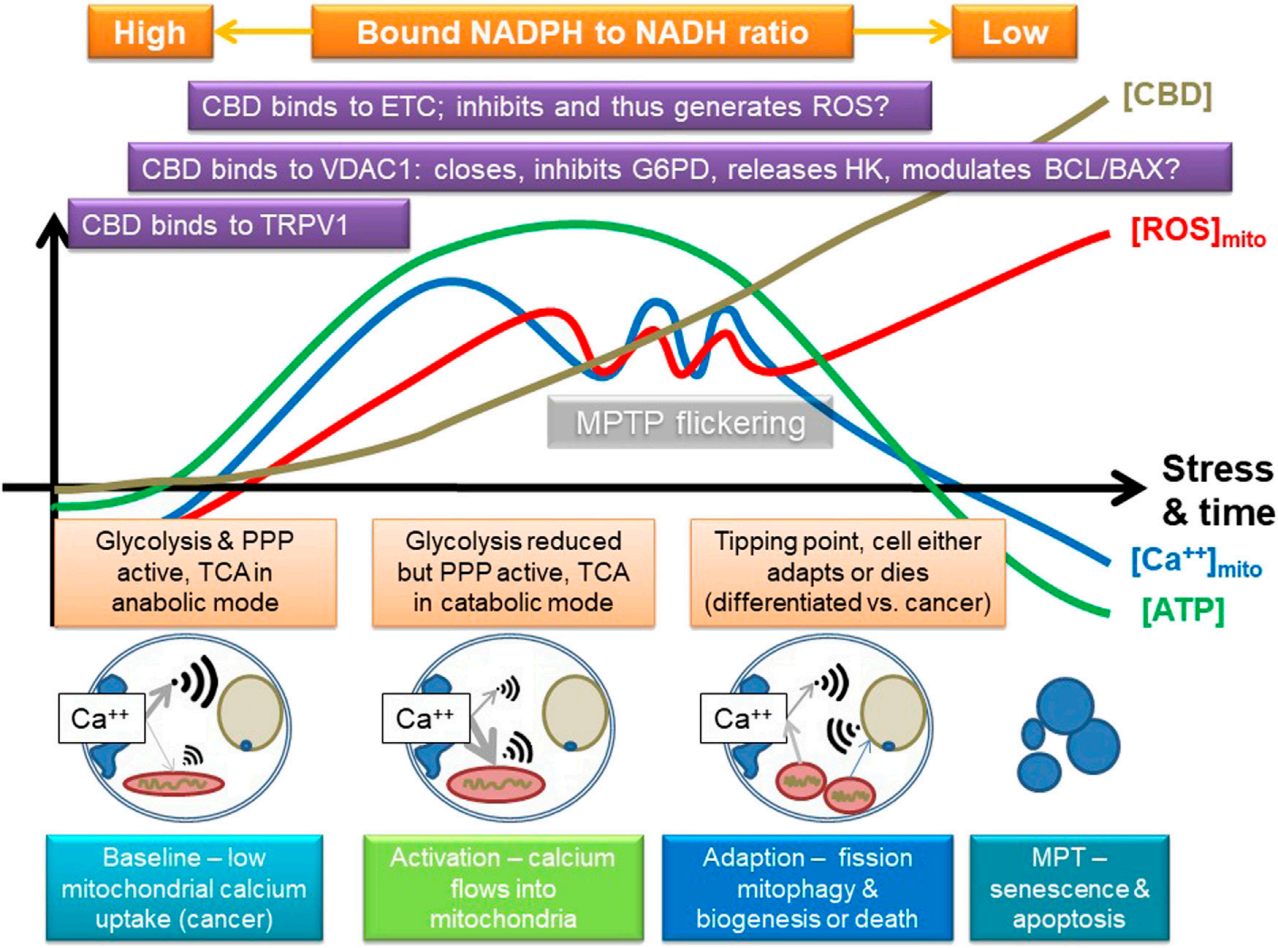
Suggested mode of action of CBD, focusing on some of its known targets, and some inferred from other phenolic compounds. CBD may stimulate calcium uptake into the cell, possibly by channels like TRPV1 and then into the mitochondria, which may involve VDAC1. It could then eventually inhibit the electron transport chain (ETC) and potentially induce the release of hexokinase (HK, which binds to VDAC1; VDAC1 is also key in controlling apoptosis *via* the BCL/BAX system), and either directly, or indirectly (e.g., by ROS), inhibits other key enzymes in glycolysis, such as G6PDH or GAPDH. This could first stimulate and then inhibit mitochondrial function, providing a powerful adaptive signal; initially, the mitochondrion can swell while it buffers calcium. However, too much calcium influx will eventually lead to overload, loss of membrane potential, and inhibition of ATP production. If the amount of calcium influx is transient, or not too large, the mitochondrion has a number of adaptive strategies, such as mitochondrial permeability transition pore flickering (MPTP) to release excessive calcium and upregulation of antioxidant pathways, which could include the pentose phosphate pathway (PPP). However, if its membrane potential starts to fall, it could start to fission and stimulate mitophagy/autophagy and initially leads to mitochondrial biogenesis and eventually leads to induction of cell death; rapid fragmentation does seem to predispose to cell death. The key effect will be to switch off anabolic growth pathways and enhance stress resistance and catabolism, which could be indicated by a shift in bound NAD(P)H. The outcome is thus likely to be concentration and cell type dependent, as well as dependent on the initial metabolic state of the cell. A key tipping point in relation to cancer is that the sudden influx of calcium will potentially increase TCA activity and potentially flow through the ETC, which will increase ROS and shift carbohydrate flux away from growth to stress resistance, for instance, by inhibiting GAPDH. However, cancer cells are generally a lot more reliant on antioxidant defense systems, as generation of ROS is a key driver of proliferation, so suddenly enhancing mitochondrial ROS is a recognized anticancer strategy as it can tip cancer cells, but not normal cells, toward cell death.

To explain this, we suggest that CBD triggers calcium uptake via TRPV and/or release from endoplasmic stores, which is then taken up by the mitochondrion. In fact, TRPV1 is also expressed intracellularly, and it has been suggested to play a role in calcium signaling to the mitochondrion (Nita et al., 2016). Combined with data that it may also modulate VDAC1 (Rimmerman et al., 2013), we suggest that as CBD is absorbed by the cell, it sequentially modulates pathways that can result in either enhanced cellular protection or, potentially, induction of cell death; key in this is both direct and indirect modulation of mitochondrial function. Support for this comes from research that indicates that CBD can induce autophagy, which could be important in epilepsy (Hosseinzadeh et al., 2016), alcohol-induced liver damage (Yang et al., 2019), and cancer (Shrivastava et al., 2011). Autophagy is pivotal in maintaining overall mitochondrial health and involves VDAC1 and mTOR; a key trigger of the process is mitochondrial dysfunction (Li et al., 2020). Induction of mitochondrial stress could lead to mitophagy, which in some cells restores homeostasis, for instance, ensuring inflammation resolution, but in others, such as cancer cells, it results in death. Figure 4 summarizes a possible mode of action. A key point here is that VDAC1 binds many different proteins, including HK and components of the Bcl2/Bax, so it is also central in controlling cell fate and a target for cancer treatment (Shoshan-Barmatz et al., 2017).

In broader terms, our data suggest that CBD, as its actions are often biphasic, could be mitohormetic (Nunn et al., 2020), inducing sublethal mitochondrial stress that results in an adaptive response via increased ROS production (Ristow and Schmeisser, 2014). In cancer cells, being at higher levels of stress than healthy cells, this tips them into a terminal oxidative stress. This selectivity has been observed following treatment of T-cells with CBD, inducing Ca^2+^ overload and apoptosis in cell lines derived from acute lymphobastic leukemia of T-lineage, but not in healthy T-cells (Olivas-Aguirre et al., 2019). This demonstrates the potential of CBD in anticancer therapy by “priming” cells, through sensitizing the mitochondria to respond better to chemotherapy and hence reducing the severity of treatment and side effects (Henley et al., 2017; Kosgodage et al., 2018).

## Conclusion

Overall, our data support a consistent narrative regarding mitochondrial modulation by CBD. Our results align with those of previous studies demonstrating that CBD induces mitochondrial membrane changes that are not too dissimilar to a reduction in the mitochondrial membrane potential and the activation of intrinsic apoptotic pathways (Shrivastava et al., 2011; Wai and Langer, 2016; Olivas-Aguirre et al., 2019). Finally, we believe that the use of 2P-FLIM could be unique in unlocking further details of how CBD works, and we aim to use the technique to study different cell types to investigate the proposed mechanism further, for example, understanding its effects on NADH, NADPH, and FAD lifetimes. In particular, we will seek evidence of biphasic effects in response to different doses across a range of timescales.

## Data Availability

The original contributions presented in the study are included in the article/Supplementary Material; further inquiries can be directed to the corresponding author.

## Supplementary Material

FIGURE S1 | **NAD(P)H autofluorescence and mitochondrial staining overlap**. Representative images of 2P-derived NAD(P)H autofluorescence and MitoTracker Red-stained MCF7 cells. Superimposed images show the NAD(P)H signal matching mitochondrial morphology.

FIGURE S2 | **Identifying mitochondrial and cytosolic pixels for analysis**. Representative 2P NAD(P)H FLIM image illustrating selection of pixels to be anaylsed.

FIGURE S3 | **2P NAD(P)H FLIM and the Goodness of Fit (Chi Squared)**. Representative 2P NAD(P)H FLIM-derived images for each treatment group, colour-coded to the obtained chi squared value (χ^2^).

## References

[B1] Adem S. Comakli V. Kuzu M. Demirdag R. (2014). Investigation of the effects of some phenolic compounds on the activities of glucose-6-phosphate dehydrogenase and 6-phosphogluconate dehydrogenase from human erythrocytes. J. Biochem. Mol. Toxicol. 28 (11), 510–514. 10.1002/jbt.21592 25130191

[B2] Alam S. R. Wallrabe H. Svindrych Z. Chaudhary A. K. Christopher K. G. Chandra D. (2017). Investigation of mitochondrial metabolic response to doxorubicin in prostate cancer cells: An NADH, FAD and tryptophan FLIM Assay. Sci. Rep. 7 (1), 10451. 10.1038/s41598-017-10856-3 28874842 PMC5585313

[B3] Armstrong J. L. Hill D. S. McKee C. S. Hernandez-Tiedra S. Lorente M. Lopez-Valero I. (2015). Exploiting cannabinoid-induced cytotoxic autophagy to drive melanoma cell death. J. Invest. Dermatol. 135, 1629. 10.1038/jid.2015.45 25674907

[B4] Baker D. Pryce G. Croxford J. L. Brown P. Pertwee R. G. Huffman J. W. (2000). Cannabinoids control spasticity and tremor in a multiple sclerosis model. Nature 404 (6773), 84–87. 10.1038/35003583 10716447

[B5] Bird D. K. Yan L. Vrotsos K. M. Eliceiri K. W. Vaughan E. M. Keely P. J. (2005). Metabolic mapping of MCF10A human breast cells via multiphoton fluorescence lifetime imaging of the coenzyme NADH. Cancer Res. 65 (19), 8766–8773. 10.1158/0008-5472.CAN-04-3922 16204046

[B6] Bisogno T. Hanuš L. De Petrocellis L. Tchilibon S. Ponde D. E. Brandi I. (2001). Molecular targets for cannabidiol and its synthetic analogues: effect on vanilloid VR1 receptors and on the cellular uptake and enzymatic hydrolysis of anandamide. Br. J. Pharmacol. 134 (4), 845–852. 10.1038/sj.bjp.0704327 11606325 PMC1573017

[B7] Blacker T. S. Duchen M. R. (2016). Investigating mitochondrial redox state using NADH and NADPH autofluorescence. Free Radic. Biol. Med. 100, 53–65. 10.1016/j.freeradbiomed.2016.08.010 27519271 PMC5145803

[B8] Blignaut M. Loos B. Botchway S. W. Parker A. W. Huisamen B. (2019). Ataxia-Telangiectasia Mutated is located in cardiac mitochondria and impacts oxidative phosphorylation. Sci. Rep. 9 (1), 4782. 10.1038/s41598-019-41108-1 30886180 PMC6423017

[B9] Bömer M. Pérez‐Salamó I. Florance H. V. Salmon D. Dudenhoffer J. Finch P. (2020). Jasmonates induce Arabidopsis bioactivities selectively inhibiting the growth of breast cancer cells through CDC6 and mTOR. New Phytol. 229 (4), 2120–2134. 10.1111/nph.17031 33124043 PMC8022592

[B10] Botchway S. W. Scherer K. M. Hook S. Stubbs C. D. Weston E. Bisby R. H. (2015). A series of flexible design adaptations to the Nikon E-C1 and E-C2 confocal microscope systems for UV, multiphoton and FLIM imaging. J. Microsc. 258, 68. 10.1111/jmi.12218 25664385

[B11] Bujak J. K. Kosmala D. Szopa I. M. Majchrzak K. Bednarczyk P. (2019). Inflammation, cancer and immunity—implication of TRPV1 channel. Front. Oncol. 9, 1087. 10.3389/fonc.2019.01087/full 31681615 PMC6805766

[B12] Chakraborty S. Nian F-S. Tsai J-W. Karmenyan A. Chiou A. (2016). Quantification of the metabolic state in cell-model of Parkinson’s disease by fluorescence lifetime imaging microscopy. Sci. Rep. 6, 19145. 10.1038/srep19145 26758390 PMC4725947

[B13] Chen H. Chan D. C. (2017). Mitochondrial dynamics in regulating the unique phenotypes of cancer and stem cells. Cell Metab. 26 (1), 39–48. 10.1016/j.cmet.2017.05.016 28648983 PMC5539982

[B14] Danese A. Patergnani S. Bonora M. Wieckowski M. R. Previati M. Giorgi C. (2017). Calcium regulates cell death in cancer: roles of the mitochondria and mitochondria-associated membranes (MAMs). Biochim. Biophys. Acta 1858 (8), 615–627. 10.1016/j.bbabio.2017.01.003 28087257

[B15] Gali-Muhtasib H. Hmadi R. Kareh M. Tohme R. Darwiche N. (2015). Cell death mechanisms of plant-derived anticancer drugs: beyond apoptosis. Apoptosis 20 (12), 1531–1562. 10.1007/s10495-015-1169-2 26362468

[B16] Gandin A. Dizengremel P. Jolivet Y. (2021). Integrative role of plant mitochondria facing oxidative stress: The case of ozone. Plant Physiol. Biochem. 159, 202–210. 10.1016/j.plaphy.2020.12.019 33385703

[B17] Giorgi C. Missiroli S. Patergnani S. Duszynski J. Wieckowski M. R. Pinton P. (2015). Mitochondria-associated membranes: Composition, molecular mechanisms, and physiopathological implications. Antioxid. Redox Signal. 22 (12), 995–1019. 10.1089/ars.2014.6223 25557408

[B18] Goldin N. Arzoine L. Heyfets A. Israelson A. Zaslavsky Z. Bravman T. (2008). Methyl jasmonate binds to and detaches mitochondria-bound hexokinase. Oncogene 27 (34), 4636–4643. 10.1038/onc.2008.108 18408762

[B19] Gorlach S. Fichna J. Lewandowska U. (2015). Polyphenols as mitochondria-targeted anticancer drugs. Cancer Lett. 366 (2), 141–149. 10.1016/j.canlet.2015.07.004 26185003

[B20] Gray R. A. Stott C. G. Jones N. A. Di Marzo V. Whalley B. J. (2020). Anticonvulsive properties of cannabidiol in a model of generalized seizure are transient receptor potential vanilloid 1 dependent. Cannabis Cannabinoid Res. 5 (2), 145–149. 10.1089/can.2019.0028 32656346 PMC7347071

[B21] Guard S. E. Chapnick D. A. Poss Z. Ebmeier C. C. Jacobsen J. Nemkov T. (2020). Multi-omic analysis reveals cannabidiol disruption of cholesterol homeostasis in human cell lines. bioRxiv. 10.1101/2020.06.03.130864. 10.1016/j.mcpro.2022.100262 PMC9525918 35753663

[B22] Gugliandolo A. Pollastro F. Bramanti P. Mazzon E. (2020). Cannabidiol exerts protective effects in an *in vitro* model of Parkinson’s disease activating AKT/mTOR pathway. Fitoterapia. 143, 104553. 10.1101/2020.06.03.130864 32184097

[B23] Guo H.-W. Yu J.-S. Hsu S.-H. Wei Y.-H. Lee O. K. Dong C.-Y. (2015). Correlation of NADH fluorescence lifetime and oxidative phosphorylation metabolism in the osteogenic differentiation of human mesenchymal stem cell. J. Biomed. Opt. 20 (1), 17004. 10.1117/1.JBO.20.1.017004 25629291

[B24] Henley A. B. Yang L. Chuang K. L. Sahuri-Arisoylu M. Wu L. H. Bligh S. W. A. (2017). Withania somnifera root extract enhances chemotherapy through “priming”. PLoS One 12 (1), e0170917. 10.1371/journal.pone.0170917 28129345 PMC5271386

[B25] Hosseinzadeh M. Nikseresht S. Khodagholi F. Naderi N. Maghsoudi N. (2016). Cannabidiol post-treatment alleviates rat epileptic-related behaviors and activates hippocampal cell autophagy pathway along with antioxidant defense in chronic phase of pilocarpine-induced seizure. J. Mol. Neurosci. 58 (4), 432–440. 10.1007/s12031-015-0703-6 26738731

[B26] Huang L. Yu L-J. Zhang X. Fan B. Wang F.-Z. Dai Y.-S. (2019). Autophagy regulates glucose-mediated root meristem activity by modulating ROS production in Arabidopsis. Autophagy 15 (3), 407–422. 10.1080/15548627.2018.1520547 30208757 PMC6351127

[B27] Huestis M. A. (2007). Human cannabinoid pharmacokinetics. Chem. Biodivers 4, 1770. 10.1002/cbdv.200790152 17712819 PMC2689518

[B28] Iannotti F. A. Hill C. L. Leo A. Alhusaini A. Soubrane C. Mazzarella E. (2014). Nonpsychotropic plant cannabinoids, cannabidivarin (CBDV) and cannabidiol (CBD), activate and desensitize transient receptor potential vanilloid 1 (TRPV1) channels *in Vitro*: potential for the treatment of neuronal hyperexcitability. ACS Chem. Neurosci. 5 (11), 1131–1141. 10.1021/cn5000524 25029033

[B29] Ibeas Bih C. Chen T. Nunn A. V. W. Bazelot M. Dallas M. Whalley B. J. (2015). Molecular targets of cannabidiol in neurological disorders. Neurotherapeutics 12 (4), 699–730. 10.1007/s13311-015-0377-3 26264914 PMC4604182

[B30] Kimata S. Mochizuki D. Satoh J. Kitano K. Kanesaki Y. Takeda K. (2018). Intracellular free flavin and its associated enzymes participate in oxygen and iron metabolism in Amphibacillus xylanus lacking a respiratory chain. FEBS Open Bio 8 (6), 947–961. 10.1002/2211-5463.12425 PMC5986008 29928575

[B31] Kis B. Ifrim F. C. Buda V. Avram S. Pavel I. Z. Antal D. (2019). Cannabidiol-from plant to human body: A promising bioactive molecule with multi-target effects in cancer. Int. J. Mol. Sci. 20 (23), 5905. 10.3390/ijms20235905 PMC6928757 31775230

[B32] Kosgodage U. S. Mould R. Henley A. B. Nunn A. V. Guy G. W. Thomas E. L. (2018). Cannabidiol (CBD) is a novel inhibitor for exosome and microvesicle (EMV) release in cancer. Front. Pharmacol. 9, 889. 10.3389/fphar.2018.00889 30150937 PMC6099119

[B33] Kroemer G. (2006). Mitochondria in cancer. Oncogene 25, 4630. 10.1038/sj.onc.1209589 16892077

[B34] Laus M. N. Soccio M. (2020). First evidence of a protective effect of plant bioactive compounds against H2O2-induced aconitase damage in durum wheat mitochondria. Antioxidants 9 (12), 1256. 10.3390/antiox9121256 PMC7763331 33321766

[B35] Li X. Zhang W. Cao Q. Wang Z. Zhao M. Xu L. (2020). Mitochondrial dysfunction in fibrotic diseases. Cell Death Discov. Internet 6 (1), 80. 10.1038/s41420-020-00316-9 PMC7474731 32963808

[B36] Marchi S. Pinton P. (2016). Alterations of calcium homeostasis in cancer cells. Curr. Opin. Pharmacol. 29, 1–6. 10.1016/j.coph.2016.03.002 27043073

[B37] Mato S. Victoria Sánchez-Gómez M. Matute C. (2010). Cannabidiol induces intracellular calcium elevation and cytotoxicity in oligodendrocytes. Glia 58 (14), 1739–1747. 10.1002/glia.21044 20645411

[B38] McAllister S. D. Soroceanu L. Desprez P. Y. (2015). The antitumor activity of plant-derived non-psychoactive cannabinoids. J. Neuroimmune Pharmacol. 10, 255. 10.1007/s11481-015-9608-y 25916739 PMC4470774

[B39] Mechoulam R. Parker L. A. Gallily R. (2002). Cannabidiol: an overview of some pharmacological aspects. J. Clin. Pharmacol. 42 (S1), 11S–19S. 10.1002/j.1552-4604.2002.tb05998.x 12412831

[B40] Nita I. I. Caspi Y. Gudes S. Fishman D. Lev S. Hersfinkel M. (2016). Privileged crosstalk between TRPV1 channels and mitochondrial calcium shuttling machinery controls nociception. Biochim. Biophys. Acta 1863 (12), 2868–2880. 10.1016/j.bbamcr.2016.09.009 27627464

[B41] Nunn A. V. Henley A. Brody L. P. Bell J. D. (2013). Phytocannabinoids modulate mitochondrial dynamics in cell lines; stress adaptation. London, United Kingdom: European Workshop on Cannabinoid Research.

[B42] Nunn A. V. W. Guy G. W. Botchway S. W. Bell J. D. (2020). From sunscreens to medicines: can a dissipation hypothesis explain the beneficial aspects of many plant compounds?. Phytotherapy Res. 34, 1868–1888. 10.1002/ptr.6654 PMC7496984 32166791

[B43] Olivas-Aguirre M. Torres-López L. Valle-Reyes J. S. Hernández-Cruz A. Pottosin I. Dobrovinskaya O. (2019). Cannabidiol directly targets mitochondria and disturbs calcium homeostasis in acute lymphoblastic leukemia. Cell Death Dis. 10 (10), 779. 10.1038/s41419-019-2024-0 31611561 PMC6791884

[B44] Oliveira M. R. d. Nabavi S. F. Daglia M. Rastrelli L. Nabavi S. M. (2016). Epigallocatechin gallate and mitochondria—a story of life and death. Pharmacol. Res. 104, 70–85. 10.1016/j.phrs.2015.12.027 26731017

[B45] O’Connell B. K. Gloss D. Devinsky O. (2017). Cannabinoids in treatment-resistant epilepsy: A review. Epilepsy Behav. 70, 341–348. 10.1016/j.yebeh.2016.11.012 28188044

[B46] Pisanti S. Malfitano A. M. Ciaglia E. Lamberti A. Ranieri R. Cuomo G. (2017). Cannabidiol: state of the art and new challenges for therapeutic applications. Pharmacol. Ther. 175, 133–150. 10.1016/j.pharmthera.2017.02.041 28232276

[B47] Popov L. (2020). Mitochondrial biogenesis: An update. J. Cell. Mol. Med. 24 (9), 4892–4899. 10.1111/jcmm.15194 32279443 PMC7205802

[B48] Rad E. Murray J. Tee A. (2018). Oncogenic signalling through mechanistic target of rapamycin (mTOR): A driver of metabolic transformation and cancer progression. Cancers 10 (1), 5. 10.3390/cancers10010005 PMC5789355 29301334

[B49] Rambold A. S. Pearce E. L. (2018). Mitochondrial dynamics at the interface of immune cell metabolism and function. Trends Immunol. 39 (1), 6–18. 10.1016/j.it.2017.08.006 28923365

[B50] Ramer R. Hinz B. (2017). Cannabinoids as anticancer drugs. Adv. Pharmacol. 80, 397–436. 10.1016/bs.apha.2017.04.002 28826542

[B51] Rasouli H. Farzaei M. H. Mansouri K. Mohammadzadeh S. Khodarahmi R. (2016). Plant cell cancer: may natural phenolic compounds prevent onset and development of plant cell malignancy? A literature review. Molecules 21 (9), 21. 10.3390/molecules21091104 PMC6274315 27563858

[B52] Redhu A. K. Bhat J. P. (2020). Mitochondrial glucose 6-phosphate dehydrogenase and 6-phosphogluconate dehydrogenase abrogate p53 induced apoptosis in a yeast model: possible implications for apoptosis resistance in cancer cells. Biochim. Biophys. Acta Gen. Subj 1864 (3), 129504. 10.1016/j.bbagen.2019.129504 31862471

[B53] Rimmerman N. Ben-Hail D. Porat Z. Juknat A. Kozela E. Daniels M. P. (2013). Direct modulation of the outer mitochondrial membrane channel, voltage-dependent anion channel 1 (VDAC1) by cannabidiol: A novel mechanism for cannabinoid-induced cell death. Cell Death Dis. 4 (12), e949. 10.1038/cddis.2013.471 24309936 PMC3877544

[B54] Ristow M. Schmeisser K. (2014). Mitohormesis: promoting health and lifespan by increased levels of reactive oxygen species (ROS). Dose Response 12, 288. 10.2203/dose-response.13-035.Ristow 24910588 PMC4036400

[B55] Rossi A. Pizzo P. Filadi R. (2019). Calcium, mitochondria and cell metabolism: A functional triangle in bioenergetics. Biochim. Biophys. Acta Mol. Cell. Res. 1866 (7), 1068–1078. 10.1016/j.bbamcr.2018.10.016 30982525

[B56] Ryan D. Drysdale A. J. Lafourcade C. Pertwee R. G. Platt B. (2009). Cannabidiol targets mitochondria to regulate intracellular Ca2+ levels. J. Neurosci. 29 (7), 2053–2063. 10.1523/JNEUROSCI.4212-08.2009 19228959 PMC6666323

[B57] Sayers T. J. (2011). Targeting the extrinsic apoptosis signaling pathway for cancer therapy. Cancer Immunol. Immunother. 60 (8), 1173–1180. 10.1007/s00262-011-1008-4 21626033 PMC11028721

[B58] Schneckenburger H. Wagner M. Weber P. Strauss W. S. Sailer R. (2004). Autofluorescence lifetime imaging of cultivated cells using a UV picosecond laser diode. J. Fluoresc 14 (5), 649–654. 10.1023/b:jofl.0000039351.09916.cc 15617271

[B59] Senft D. Ronai Z. A. (2016). Regulators of mitochondrial dynamics in cancer. Curr. Opin. Cell Biol. 39, 43–52. 10.1016/j.ceb.2016.02.001 26896558 PMC4828329

[B60] Sharick J. T. Favreau P. F. Gillette A. A. Sdao S. M. Merrins M. J. Skala M. C. (2018). Protein-bound NAD(P)H lifetime is sensitive to multiple fates of glucose carbon. Sci. Rep. 8 (1), 5456. 10.1038/s41598-018-23691-x 29615678 PMC5883019

[B61] Sharma A. Smith H. J. Yao P. Mair W. B. (2019). Causal roles of mitochondrial dynamics in longevity and healthy aging. EMBO Rep. 20 (12), e48395. 10.15252/embr.201948395 31667999 PMC6893295

[B62] Shin E. S. Park J. Shin J-M. Cho D. Cho S. Y. Shin D. W. (2008). Catechin gallates are NADP+-competitive inhibitors of glucose-6-phosphate dehydrogenase and other enzymes that employ NADP+ as a coenzyme. Bioorg. Med. Chem. 16 (7), 3580–3586. 10.1016/j.bmc.2008.02.030 18313308

[B63] Shoshan-Barmatz V. De S. Meir A. (2017). The mitochondrial voltage-dependent anion channel 1, Ca2+ transport, apoptosis, and their regulation. Front. Oncol. 7, 60. 10.3389/fonc.2017.00060/full 28443244 PMC5385329

[B64] Shoshan-Barmatz V. Krelin Y. Shteinfer-Kuzmine A. (2018). VDAC1 functions in Ca2+ homeostasis and cell life and death in health and disease. Cell Calcium 69, 81–100. 10.1016/j.ceca.2017.06.007 28712506

[B65] Shrivastava A. Kuzontkoski P. M. Groopman J. E. Prasad A. (2011). Cannabidiol induces programmed cell death in breast cancer cells by coordinating the cross-talk between apoptosis and autophagy. Mol. Cancer Ther. 10 (7), 1161–1172. 10.1158/1535-7163.MCT-10-1100 21566064

[B66] Singer E. Judkins J. Salomonis N. Matlaf L. Soteropoulos P. McAllister S. (2015). Reactive oxygen species-mediated therapeutic response and resistance in glioblastoma. Cell Death Dis. 6, e1601. 10.1038/cddis.2014.566 25590811 PMC4669764

[B67] Skala M. C. Riching K. M. Bird D. K. Gendron-Fitzpatrick A. Eickhoff J. Eliceiri K. W. (2007a). In vivo multiphoton fluorescence lifetime imaging of protein-bound and free nicotinamide adenine dinucleotide in normal and precancerous epithelia. J. Biomed. Opt. 12 (2), 024014. 10.1117/1.2717503 17477729 PMC2743958

[B68] Skala M. C. Riching K. M. Gendron-Fitzpatrick A. Eickhoff J. Eliceiri K. W. White J. G. (2007b). *In vivo* multiphoton microscopy of NADH and FAD redox states, fluorescence lifetimes, and cellular morphology in precancerous epithelia. Proc. Natl. Acad. Sci. U.S.A. 104 (49), 19494–19499. 10.1073/pnas.0708425104 18042710 PMC2148317

[B69] Stevens J. F. Revel J. S. Maier C. S. (2018). Mitochondria-centric review of polyphenol bioactivity in cancer models. Antioxid. Redox Signal. 29 (16), 1589. 10.1089/ars.2017.7404 29084444 PMC6207154

[B70] Stringari C. Cinquin A. Cinquin O. Digman M. A. Donovan P. J. Gratton E. (2011). Phasor approach to fluorescence lifetime microscopy distinguishes different metabolic states of germ cells in a live tissue. Proc. Natl. Acad. Sci. U.S.A 108 (33), 13582–13587. 10.1073/pnas.1108161108 21808026 PMC3158156

[B71] Suhling K. Hirvonen L. M. Levitt J. A. Chung P.-H. Tregidgo C. Le Marois A. (2015). Fluorescence lifetime imaging (FLIM): Basic concepts and some recent developments. Med. Photon. 27, 3–40. 10.1016/j.medpho.2014.12.001

[B72] Sun S. Hu F. Wu J. Zhang S. (2017). Cannabidiol attenuates OGD/R-induced damage by enhancing mitochondrial bioenergetics and modulating glucose metabolism via pentose-phosphate pathway in hippocampal neurons. Redox Biol. 11, 577–585. 10.1016/j.redox.2016.12.029 28110213 PMC5247568

[B73] Tapia P. C. (2006). Sublethal mitochondrial stress with an attendant stoichiometric augmentation of reactive oxygen species may precipitate many of the beneficial alterations in cellular physiology produced by caloric restriction, intermittent fasting, exercise and dietary phytonutrients: “Mitohormesis” for health and vitality. Med. Hypotheses 66 (4), 832–843. 10.1016/j.mehy.2005.09.009 16242247

[B74] Tewari D. Ahmed T. Chirasani V. R. Singh P. K. Maji S. K. Senapati S. (2015). Modulation of the mitochondrial voltage dependent anion channel (VDAC) by curcumin. Biochim. Biophys. Acta 1848, 151–158. 10.1016/j.bbamem.2014.10.014 25459681

[B75] Tewari D. Majumdar D. Vallabhaneni S. Bera A. K. (2017). Aspirin induces cell death by directly modulating mitochondrial voltage-dependent anion channel (VDAC). Sci. Rep. 7 (1), 45184. 10.1038/srep45184 28327594 PMC5361111

[B76] Trigos A. S. Pearson R. B. Papenfuss A. T. Goode D. L. (2018). How the evolution of multicellularity set the stage for cancer. Br. J. Cancer 118 (2), 145–152. 10.1038/bjc.2017.398 29337961 PMC5785747

[B77] Valente A. J. Maddalena L. A. Robb E. L. Moradi F. Stuart J. A. (2017). A simple ImageJ macro tool for analyzing mitochondrial network morphology in mammalian cell culture. Acta Histochem. 119 (3), 315–326. 10.1016/j.acthis.2017.03.001 28314612

[B78] Wai T. Langer T. (2016). Mitochondrial dynamics and metabolic regulation. Trends Endocrinol. Metab. 212 (4), 379–387. 10.1016/j.tem.2015.12.001 26754340

[B79] Wallace D. C. (2012). Mitochondria and cancer. Nat. Rev. Cancer 12 (10), 685–698. 10.1038/nrc3365 23001348 PMC4371788

[B80] Wei Y. Zhang Y.-J. Cai Y. Xu M.-H. (2015). The role of mitochondria in mTOR-regulated longevity. Biol. Rev. Camb. Philos. Soc. 90 (1), 167–181. 10.1111/brv.12103 24673778

[B81] Wu H.-Y. Huang C.-H. Lin Y.-H. Wang C.-C. Jan T.-R. (2018). Cannabidiol induced apoptosis in human monocytes through mitochondrial permeability transition pore-mediated ROS production. Free Radic. Biol. Med. 124, 311–318. 10.1016/j.freeradbiomed.2018.06.023 29940353

[B82] Yang L. Yang C. Thomes P. G. Kharbanda K. K. Casey C. A. McNiven M. A. (2019). Lipophagy and alcohol-induced fatty liver. Front. Pharmacol. 10, 495. 10.3389/fphar.2019.00495/full 31143122 PMC6521574

[B83] Yaseen M. A. Sutin J. Wu W. Fu B. Uhlirova H. Devor A. (2017). Fluorescence lifetime microscopy of NADH distinguishes alterations in cerebral metabolism *in vivo* . Biomed. Opt. Express 8 (5), 2368–2385. 10.1364/BOE.8.002368 28663879 PMC5480486

[B84] Yu Q. Heikal A. A. (2009). Two-photon autofluorescence dynamics imaging reveals sensitivity of intracellular NADH concentration and conformation to cell physiology at the single-cell level. J. Photochem. Photobiol. B. 95 (1), 46–57. 10.1016/j.jphotobiol.2008.12.010 19179090 PMC2739809

