# Supplementary figures and images for: Cannabidiol Modulates Mitochondrial Redox and Dynamics in MCF7 Cancer Cells: A Study Using Fluorescence Lifetime Imaging Microscopy of NAD(P)H
Source: Front Mol Biosci. 2021 May 11;8:630107. doi: 10.3389/fmolb.2021.630107 (PMC8144465; doi:10.3389/fmolb.2021.630107)

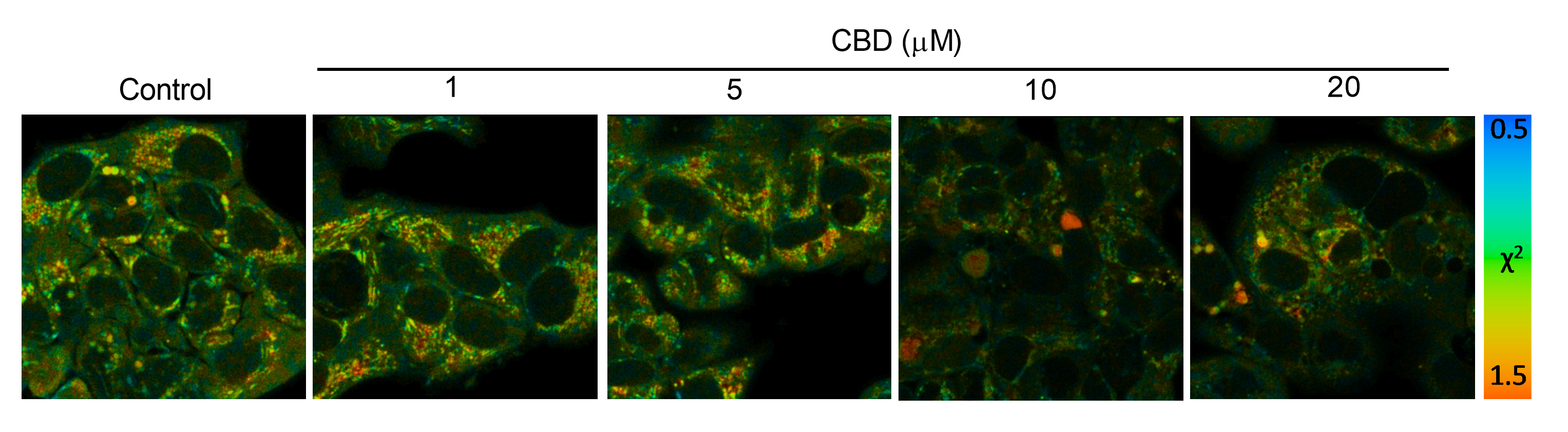

Supplement: Supplementary file 1 [file Image3.tif]

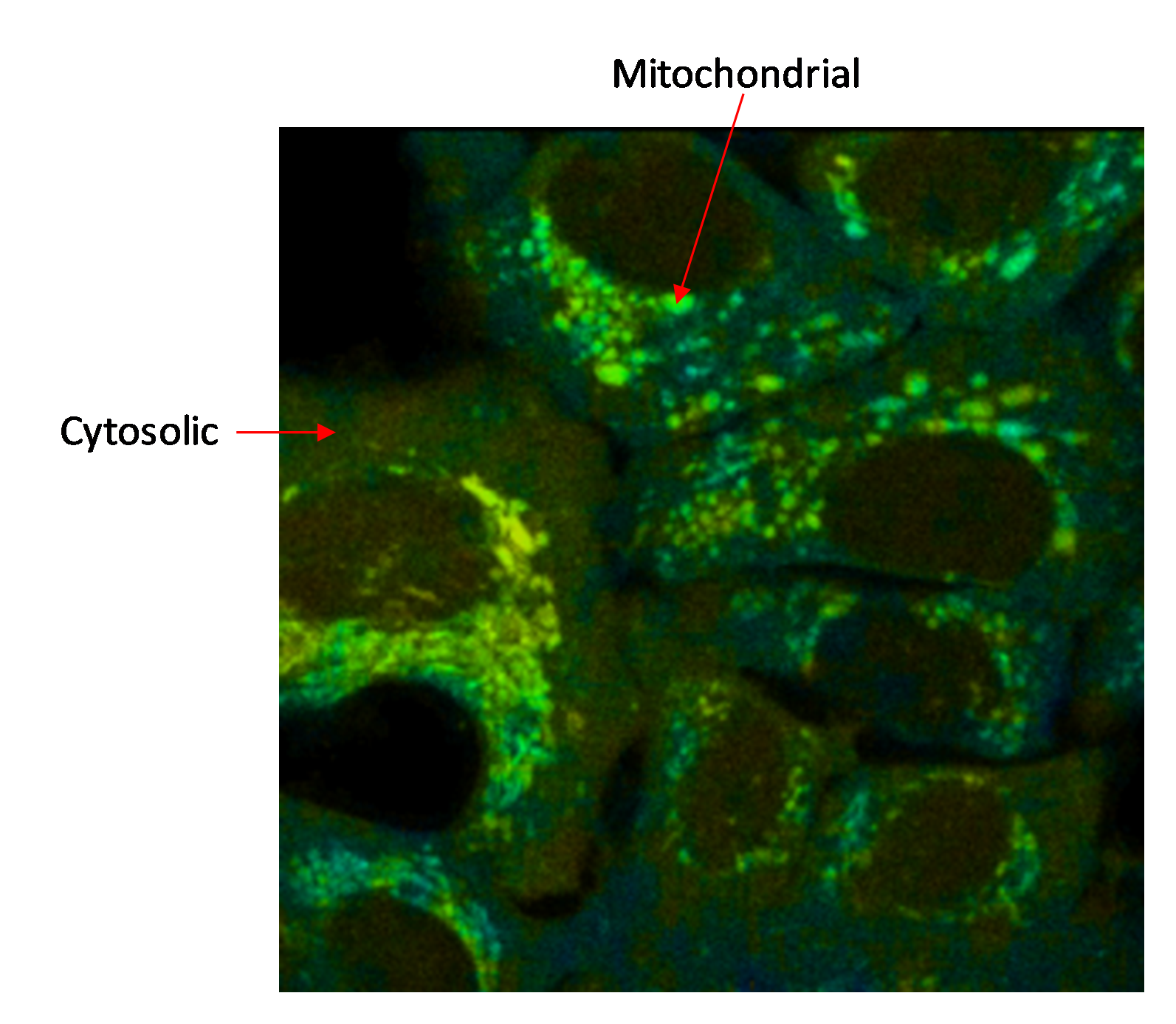

Supplement: Supplementary file 2 [file Image2.tif]

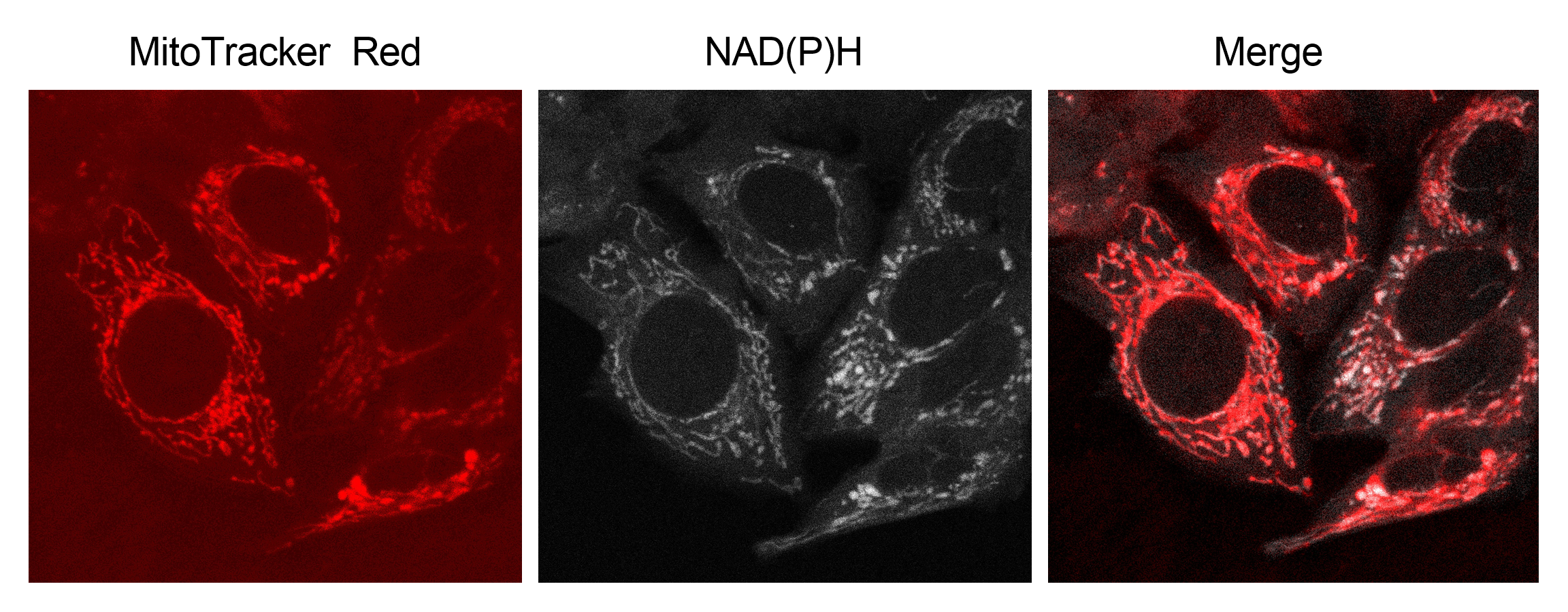

Supplement: Supplementary file 3 [file Image1.tif]
